# Diversified Regulation of Cytokinin Levels and Signaling During *Botrytis cinerea* Infection in *Arabidopsis*

**DOI:** 10.3389/fpls.2021.584042

**Published:** 2021-02-10

**Authors:** Beibei Li, Ruolin Wang, Shiya Wang, Jiang Zhang, Ling Chang

**Affiliations:** ^1^State Key Laboratory of Biocatalysis and Enzyme Engineering, School of Life Sciences, Hubei University, Wuhan, China; ^2^School of Biology and Agricultural Resources, Huanggang Normal University, Huanggang, China

**Keywords:** cytokinins, *Botrytis cinerea*, plant immunity, jasmonic acid, ethylene, hormonal crosstalk

## Abstract

Cytokinins (CKs) can modulate plant immunity to various pathogens, but how CKs are involved in plant defense responses to the necrotrophic pathogen *Botrytis cinerea* is still unknown. Here, we found that *B. cinerea* infection induced transcriptional changes in multiple genes involved in the biosynthesis, degradation, and signaling of CKs, as well as their contents, in pathogen-infected *Arabidopsis* leaves. Among the CKs, the gene expression of *CYTOKININ OXIDASE/DEHYDROGENASE 5* (*CKX5*) was remarkably induced in the local infected leaves and the distant leaves of the same plant without pathogen inoculation. *Cis*-zeatin (*c*Z) and its riboside (*c*ZR) accumulated considerably in infected leaves, suggesting an important role of the *cis*-zeatin type of CKs in the plant response to *B. cinerea*. Cytokinin double-receptor mutants were more susceptible to *B. cinerea* infection, whereas an exogenous CK treatment enhanced the expression levels of defense-related genes and of jasmonic acid (JA) and ethylene (ET), but not salicylic acid (SA), resulting in higher resistance of *Arabidopsis* to *B. cinerea*. Investigation of CK responses to *B. cinerea* infection in the JA biosynthesis mutant, *jar1-1*, and ET-insensitive mutant, *ein2-1*, showed that CK signaling and levels of CKs, namely, those of isopentenyladenine (iP), isopentenyladenine riboside (iPR), and *trans*-zeatin (*t*Z), were enhanced in *jar1-1*-infected leaves. By contrast, reductions in iP, iPR, *t*Z, and *t*Z riboside (*t*ZR) as well as *c*ZR contents occurred in *ein2-1*-infected leaves, whose transcript levels of CK signaling genes were likewise differentially regulated. The *Arabidopsis Response Regulator 5* (*ARR5*) gene was upregulated in infected leaves of *ein2-1* whereas another type-A response regulator, *ARR16*, was significantly downregulated, suggesting the existence of a complex regulation of CK signaling via the ET pathway. Accumulation of the *cis*-zeatin type of CKs in *B. cinerea*-infected leaves depended on ET but not JA pathways. Collectively, our findings provide evidence that CK responds to *B. cinerea* infection in a variety of ways that are differently modulated by JA and ET pathways in *Arabidopsis*.

## Introduction

To detect and defend themselves against phytopathogens in their environment, plants have evolved a sophisticated innate immune system consisting of multiple layers, which are highly interconnected and tightly regulated (Jones and Dangl, 2006). The innate immunity depends on extensive transcriptional reprogramming in the host, which is activated and controlled by plant hormones, including jasmonic acid (JA), ethylene (ET), and salicylic acid (SA) (Li et al., 2019). Generally, SA is considered to play a central role in defense against biotrophic and hemi-biotrophic pathogens, while JA and ET mainly contribute to host immunity to necrotrophic pathogens including *Botrytis cinerea* (Glazebrook, 2005; AbuQamar et al., 2017).

Cytokinins (CKs), well known for their functions in controlling plant growth and development, can also affect plant resistance to disease (Pertry et al., 2009; Naseem et al., 2014). Many (hemi)biotrophic pathogens can secrete CKs or cause a local increase of cytokinin production in a host plant, to modulate host immunity and optimize nutrient supply (Spallek et al., 2018). In addition, host-derived CKs can also influence plant immunity to pathogens that do not secrete CKs by interacting with the SA pathway or by increasing the synthesis of antimicrobial phytoalexin independently of SA signaling (Choi et al., 2010; Grosskinsky et al., 2011). Yet the role of CK in plant immunity to necrotrophic pathogens such as *B. cinerea*, which does not produce detectable CKs (Cooper and Ashby, 1998; Walters and McRoberts, 2006), is far less understood. Expression of the CK biosynthesis gene *IPT* from *Agrobacterium* under the control of the *SAG12* (senescence-specific gene) promoter resulted in increased resistance of *Arabidopsis thaliana* to *B. cinerea* infection (Swartzberg et al., 2008). Arabidopsis histidine kinase 5 (AHK5), a member of the HK family of the two-component systems which involve cytokinin receptors (AHK2, AHK3, and AHK4), contributes to resistance to *B. cinerea* (Pham et al., 2012), suggesting the function of histidine kinases in regulating resistance against fungal infection. Moreover, transgenic *Nicotiana attenuata* having increased CK levels had an increased accumulation of JA metabolites (Schäfer et al., 2013). Recently, Gupta et al. (2020) revealed how CK promotes the resistance of tomato to *B. cinerea* through an SA- and ET-dependent mechanism. These cases provide evidence suggesting the potential contribution of CK to a plant defense response to *B. cinerea*, as well as the existence of cross talk between CKs and other phytohormone pathways, including those of JA, ET, and SA.

CK metabolism and signaling pathways in *Arabidopsis* are well elucidated (Kieber and Schaller, 2014). Briefly, CKs’ biosynthesis is initiated as a rate-limiting step by isopentenyl transferases (IPTs). The CK riboside 5’-monophosphate phosphoribohydrolase LONELY GUY (LOG) is responsible for the final step of CKs’ biosynthesis, in forming active CKs, such as isopentenyladenine (iP), *trans*-zeatin (*t*Z), *cis*-zeatin (*c*Z), and dihydrozeatin (DHZ), and their respective ribosides (∼R) (Kuroha et al., 2009). These CKs, however, can be inactivated by CK OXIDASE/DEHYDROGENASE (CKX). The CK signal is perceived by specific receptors named ARABIDOPSIS HISTIDINE KINASE 2 (AHK2), AHK3, and AHK4/CYTOKININ RESPONSE 1 (CRE1) (Yamada et al., 2001) and subsequently transmitted by Arabidopsis histidine phosphotransfer proteins (AHPs) to nuclear-localized type-B response regulators (type-B ARRs) that essentially function as transcription factors (Mason et al., 2005; Hutchison et al., 2006). The type-A response regulators, targets of type-B ARRs, are thus negative feedback regulators of the CK pathway (To et al., 2004).

Here we analyzed the role of CKs in the *Arabidopsis*–*B. cinerea* interactions to understand (i) whether the CK pathway and CKs’ contents are altered during *B. cinerea* infection; (ii) whether changes in CK levels may affect *Arabidopsis* resistance to *B. cinerea*; and (iii) whether the CK response to *B. cinerea* infection in plant is regulated by JA/ET pathways. Our results demonstrate that CK signaling and levels respond to *B. cinerea* infection and are under the control of JA and ET pathways.

## Materials and Methods

### Plant Material and Growth Conditions

All *Arabidopsis thaliana* plants used in this study were of the Columbia-0 (Col-0) ecotype. Seeds of the cytokinin double receptor mutants (*cre1 ahk3*, *cre1 ahk2*; Chang et al., 2015), *ARR5:GUS* and *CKX5:GUS* (Chang et al., 2015), were obtained from Dr. Thomas Schmülling (Freie Universität Berlin, Germany). Both the jasmonate-amino acid synthetase mutant *JA-resistant 1* (*jar1-1*) (Staswick et al., 2002) and the ethylene signal mutant *ethylene insensitive 2* (*ein2-1*) (Guzmán and Ecker, 1990) were kindly provided by Dr. Shunping Yan (Huazhong Agricultural University). Seeds of *Arabidopsis* plants used for *B. cinerea* infection experiments were surface-sterilized and sown on plates with solid Murashige and Skoog (MS) medium and stratified at 4°C for 3 days in the dark before germination. Then, the plates were transferred to the growth chamber. Two weeks later, the seedlings were transplanted to sterile soil to avoid infection from unspecified pathogens. The plants were grown in designated cabinets (CIMO, QHX-300BSH-III, Shanghai) under conditions of 12 h’ white light (∼100 μmol m^–2^ s^–1^) at 23°C/12 h’ darkness at 20°C, with 60% humidity.

### Pathogen Bioassays

The *B. cinerea* strain B05.10 was cultivated on potato dextrose agar (PDA) medium (Coolaber, Beijing) at 22°C for 10 days. Spores were collected, filtered, and resuspended in half-strength potato dextrose broth (1/2 PDB), to a final concentration of 2.5 × 10^5^ spores mL^–1^. For droplet inoculations, 4 μL of spore suspension was applied to single leaves of 4-weeks-old intact plants; three leaves (5th to 7th rosette leaves) were infected of each plant. Leaves were excised from plants only for rating symptoms’ severity. For the mock treatment (i.e., the control), the same amount of 1/2 PDB alone was used. After the inoculations, all the plants were kept under sealed transparent hoods at high humidity.

Two days after *B. cinerea* infection, the hoods were removed and the symptoms on the infected leaves were analyzed. Lesion diameters on leaves were measured with Image J software^1^. Following Van Wees et al. (2013), disease symptoms on inoculated leaves were recorded and grouped into four classes: lesion diameter < 2 mm (class I), 2-mm lesion with chlorosis (class II), 2–4 mm lesion with chlorosis (class III), and lesion with a spread > 4 mm (class IV). Quantification of fungal biomass relative to plant biomass by qPCR was performed as already described by Gachon and Saindrenan (2004). The abundance of *cutinase A* was quantified in the infected samples and normalized against the *Arabidopsis actin2* gene. Primers used for used for *B. cinerea* growth biomass are listed in Supplementary Table S4.

### Trypan Blue and GUS (β-Glucuronidase) Staining of Plant Materials

To visualize fungal tissue and dying plant cells, the leaves were stained with trypan blue as described before (Argueso et al., 2012). The histochemical detection of GUS activity was carried out following the methodology of Chang et al. (2013, 2015).

### Quantitative Real-Time Reverse Transcription PCR (qRT-PCR)

Total RNA was extracted from the whole leaves of plants that were either *B. cinerea*-infected or mock-treated, by using the RNAiso Plus kit (Takara, Beijing, China) and following the manufacturer’s instructions. Quality and integrity of RNA were assessed by gel electrophoresis and A_260_/A_280_ and A_260_/A_230_ ratios in a NanoDrop photometer (PeqLab, Germany). Pure and highly intact RNA samples were used for cDNA synthesis with Hifair^TM^ 1st Stranded cDNA Synthesis SuperMix for qPCR (gDNA digester plus) kit (Yeasen, Shanghai, China). The generated cDNA was then subjected to quantitative PCR (qPCR) with gene-specific primers (Supplementary Table S3), by using the TB Green^®^ Premix Ex Taq^TM^ II (Tli RNaseH Plus) kit (Takara). The qPCRs were implemented in a CFX Connect Real-Time System (Bio-Rad, United States), with three technical replicates in the same run with at least three biological replicates used. For normalization purposes, *EXP* (At4g26410) was used as an endogenous reference gene because it has high expression stability under varying plant stress conditions (Czechowski et al., 2005; Liu S. et al., 2017). Primers used for reference genes and genes of interest are listed in Supplementary Table S3. The ΔΔCt method (Livak and Schmittgen, 2001) was used to calculate the relative expression levels of genes.

### Hormone Treatment

For kinetin’s application, it was dissolved in 0.1 N sodium hydroxide (NaOH), to make a stock solution. The leaves of 4-weeks-old *Arabidopsis* plants were sprayed with a solution of 0.015% (vol/vol) Silwet L-77 (GE Healthcare, Beijing, China) containing the indicated concentrations of kinetin (Biosharp, Shanghai, China) for 3 days, three times per day. The plants were covered with transparent hoods immediately after their spraying, to retain the humidity. Mock treatments were sprayed with a solution containing only 0.015% (vol/vol) Silwet L-77.

### Quantification of Cytokinins (CKs)

After the *B. cinerea* and mock treatments were completed, the leaves were collected and frozen in liquid nitrogen. Their CKs were then measured as described previously (Liu et al., 2010).

## Results

### Regulation of CK-Related Genes by *B. cinerea* Infection

**FIGURE 1:**
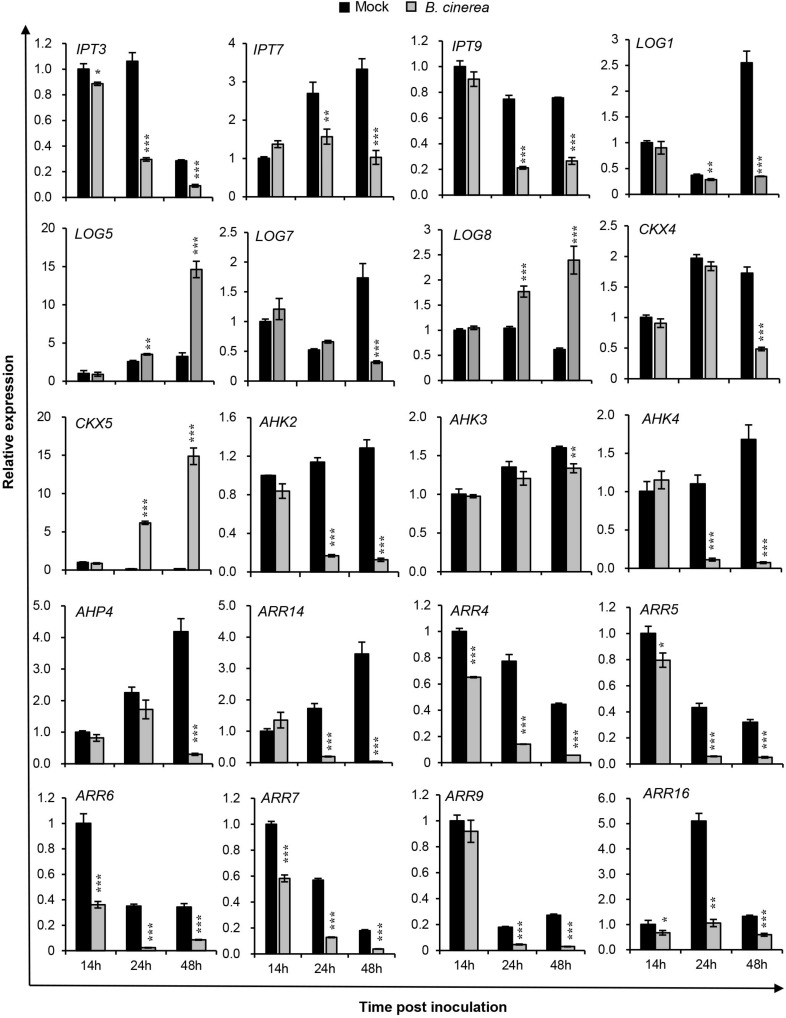
Expression profiles of selected cytokinin-related genes in *Arabidopsis* wild-type (Col-WT) plants after *Botrytis cinerea* inoculation. Transcript accumulations of cytokinin metabolism and signaling genes were determined by qRT-PCR in 4-weeks-old WT leaves inoculated with *B. cinerea* at different time-points, and likewise in corresponding leaves receiving the same amount of 1/2 PDB solution as the mock treatment. Expression levels in the mock leaves at 14 h were set to a value of 1. All data were normalized to the expression of *EXP* (At4g26410). Asterisks indicate significant differences between *B. cinerea* and mock-treated samples at the same time-point (two-tailed Student’s *t*-test: *P < 0.05, **P < 0.01, ***P < 0.001). Error bars are standard deviations (*n* = 3). Three independent experiments were performed with a similar outcome; results from one representative experiment are shown.

According to the transcriptomic dataset from the microarray platform GENEVESTIGATOR^2^, CK-related genes were transcriptionally regulated after *B. cinerea* inoculation (Supplementary Figure S1). To confirm these changes, those highly regulated CK genes identified from the available microarray data were selected and analyzed via qRT-PCR for infected leaves at 14, 24, and 48 h post inoculation (hpi) of *B. cinerea* (Figure 1). The fungus and dying plant cells at the time-points 0, 12, 14, 24, and 48 hpi were detected with trypan blue (Supplementary Figure S2); spotty blue staining, indicative of dying cells, was visible at 12 hpi and became more pronounced at 14 hpi on infected leaves. The blue staining expanded rapidly, forming a much bigger lesion on leaves at 24 hpi. By 48 hpi, the fungus had grown more aggressively, with nearly half of each leaf now necrotic.

Figure 1 shows the transcript levels of selected CK genes involved in CK metabolism and signaling components that were differentially regulated by *B. cinerea* infection vs. the mock-treated leaves. Evidently, transcript levels of CK biosynthesis genes *IPT3*, *IPT7*, *IPT9*, *LOG1*, and *LOG7* were strongly repressed from 24 hpi onward, while *LOG5* and *LOG8* expression levels were upregulated. For *CKX4*, a CK degradation enzyme gene, its expression was unchanged at 14 and 24 hpi, but reduced at 48 hpi; however, another CK degradation enzyme gene, *CKX5*, was significantly upregulated after *B. cinerea* inoculation, at levels 37-fold and 86-fold higher than mock treatments at 24 and 48 hpi, respectively. All three CK receptor genes were reduced in their expression. Compared with *AHK3*, the suppressed levels of *AHK2* and *CRE1/AHK4* were more pronounced following the *B. cinerea* treatment, suggesting their importance in the plant response to infection by this pathogen. The CK signaling genes—*AHP4*, the type-B response regulator gene *ARR14*, and the type-A Arabidopsis response regulator genes *ARR4*, *ARR5*, *ARR6*, *ARR7*, *ARR9*, and *ARR16*—were strongly repressed in the 48-h period since the *B. cinerea* inoculation. Among them, *ARR4*, *ARR6*, and *ARR16* were regulated more rapidly, being strikingly suppressed at 14 hpi. These qRT-PCR results suggested that *B. cinerea* infection strongly affects CK levels in *Arabidopsis* plants.

**FIGURE 2:**
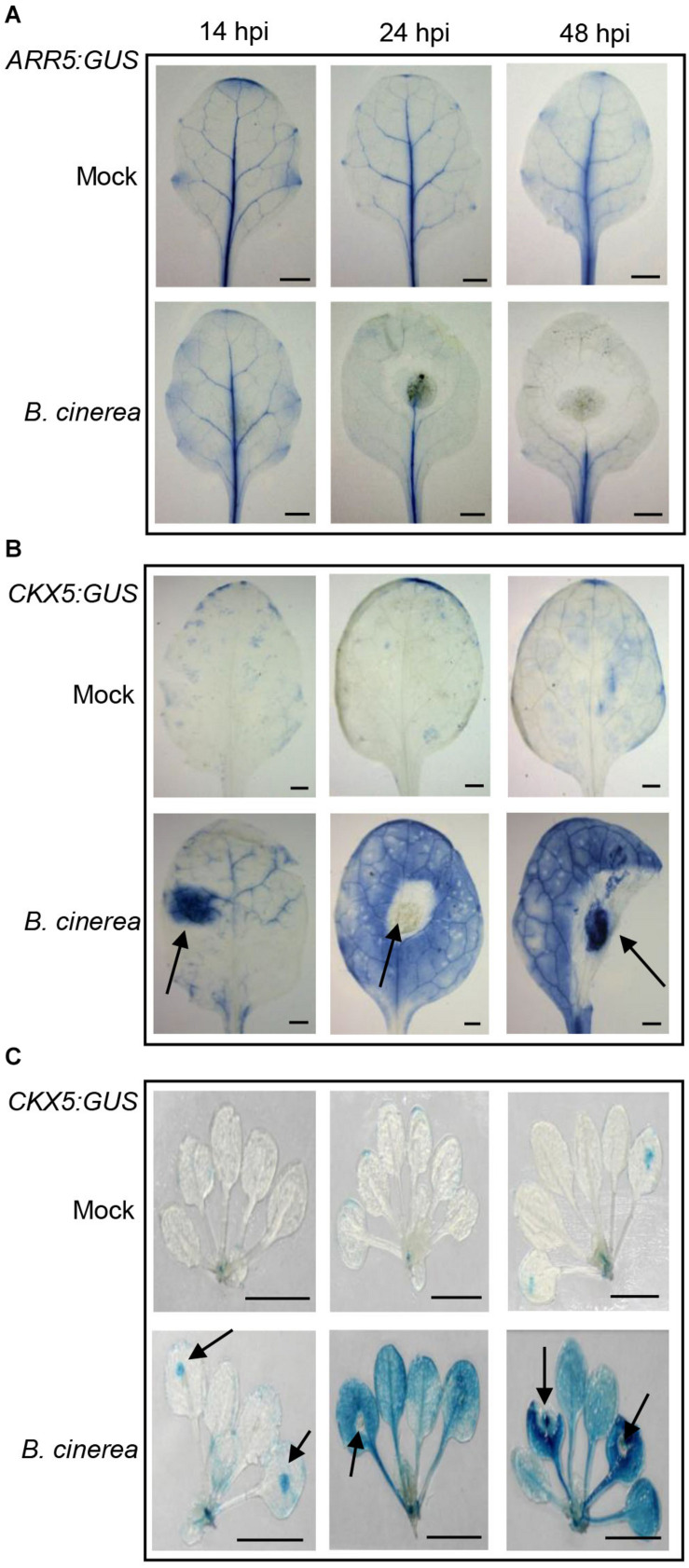
Expression of *ARR5:GUS* and *CKX5:GUS* after *Botrytis cinerea* inoculation. Expression of *ARR5:GUS* **(A)** and *CKX5:GUS* **(B)** in infected leaves at different time-points (hours) following the drop-inoculation (hpi) with *B. cinerea*. For mock treatment, the same amount of 1/2 PDB solution was applied. **(C)** Expression of *CKX5:GUS* in whole shoots after *B. cinerea* infection. Representative images are shown. For each treatment at each time-point, at least 10 plants were analyzed; 4 μL of 2.5 × 10^5^ spores mL^–1^ of *B. cinerea* were applied to the 5th–7th leaves of each plant. Leaves were detached from the shoot for photographing. The arrowheads in **(B,C)** point to the inoculation sites of *B. cinerea*. Scale bars = 1 cm.

Next we employed two reporter lines, *ARR5:GUS* (β-glucuronidase) and *CKX5:GUS*, to verify the expression pattern of *ARR5* and *CKX5* in leaves infected with *B. cinerea* at different time-points (Figure 2). As shown in Figure 2A, in the mock-treated leaves, the activity of *ARR5:GUS* was mainly detected in their vascular bundle; at 14 hpi, the *ARR5:GUS* expression was still similar to mock-treated leaves. At 24 and 48 hpi, however, GUS activity clearly diminished in the leaf portion without necrosis, further confirming the repressed CK signaling in *B. cinerea*-infected leaves. For *CKX5:GUS*, very weak expression was observed in either the mock leaves (Figure 2B) or mock seedlings (Figure 2C), but *CKX5:GUS* expression was remarkably induced at the site in the leaves where *B. cinerea* was inoculated (indicated with arrowheads in Figures 2B,C). Later, at 24 and 48 hpi, GUS activity was very strong and appeared in nearly the entire leaf except its necrotic parts (Figure 2B). Besides those leaves inoculated with *B. cinerea*, *CKX5:GUS* also showed very high expression in the distant leaves of the same plant that were not inoculated (Figure 2C), indicating that CKs’ degradation by CKX5 occurred in a systemic manner following *B. cinerea* infection.

### CK Levels Are Regulated by *B. cinerea* Infection

**FIGURE 3:**
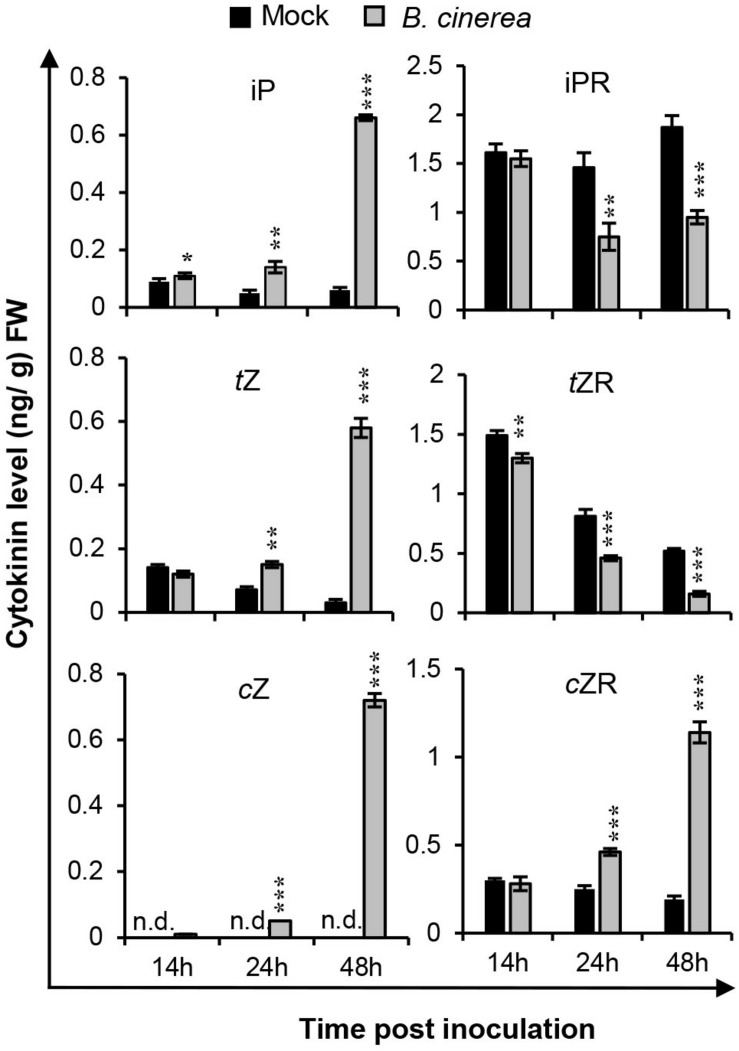
Cytokinin levels were changed after *Botrytis cinerea* infection. Isopentenyladenine (iP), isopentenyladenosine (iPR), *trans*-zeatin (*t*Z), *trans*-zeatin riboside (*t*ZR), *cis*-zeatin (cZ), and *cis*-zeatin riboside (*c*ZR) levels were measured in leaves of 4-weeks-old wild-type plants at different time-points after the *B. cinerea* inoculation or 1/2 PDB treatment (mock). Significant differences between *B. cinerea* and mock-treated samples at the same time-point were analyzed by two-tailed Student’s *t*-test. *P < 0.05; **P < 0.01; ***P < 0.001. Error bars are standard deviations. Three independent experiments were performed with similar results. FW, fresh weight.

To characterize how the endogenous cytokinin production could be affected during *B. cinerea* infection, the biological active CKs—isopentenyladenine (iP), *trans*-zeatin (*t*Z), *cis*-zeatin (*c*Z), and their ribosides (iPR, *t*ZR, *c*ZR)—were quantified for leaves inoculated with *B. cinerea* at indicated time-points (Figure 3 and Supplementary Table S1). As Figure 3 shows, compared with mock treatments, the iP content was rapidly induced to higher level in leaves post-*B. cinerea* inoculation. By contrast, the content of riboside-type iPR was significantly reduced at both 24 and 48 hpi. Similarly, the *t*Z concentration increased considerably in *B. cinerea*-infected leaves whereas the amount of *t*ZR quickly decreased after *B. cinerea* infection. However, unlike the iP and *t*Z types, both *c*Z and *c*ZR levels were dramatically elevated at 24 and 48 hpi. In mock-treated leaves, the level of *c*Z was too low to be detected, but it did reach ca. 0.72 ng/g fresh weight of *B. cinerea*-infected leaves at 48 hpi. Concerning *c*ZR, the *B. cinerea* application increased its levels by approximately 85% at 24 hpi and 500% at 48 hpi. Compared with the iP and *t*Z types, the sum of *c*Z and *c*ZR in *B. cinerea*-infected leaves rose to 10 times that of mock treated leaves by 48 hpi. The prominent alteration of *c*Z-type CK levels suggested a crucial role of *c*Z-type CK in the interaction between *Arabidopsis* and *B. cinerea*.

### Cytokinin Status Affects *Arabidopsis* Sensitivity to *B. cinerea*

**FIGURE 4:**
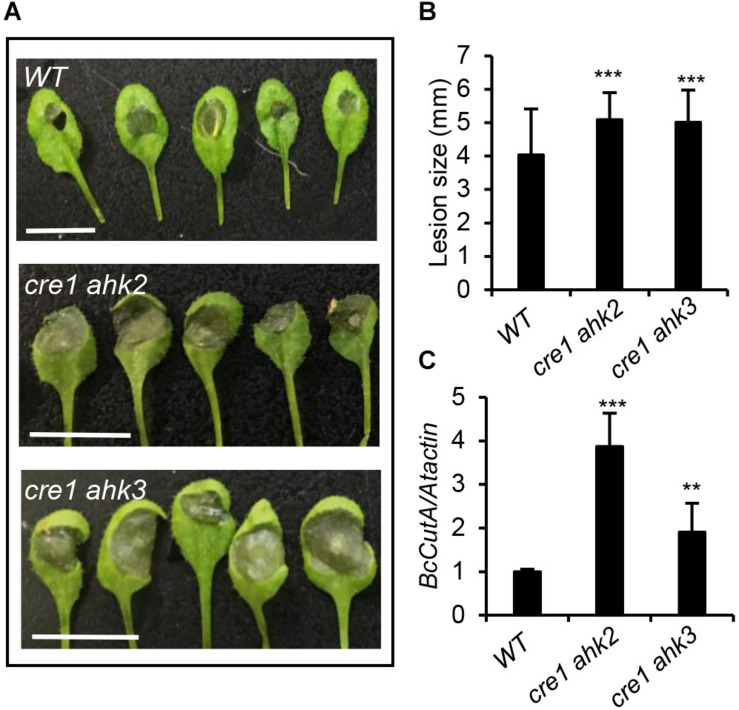
Cytokinin double-receptor mutants were more susceptible to *Botrytis cinerea* infection. **(A)** Disease symptoms in 4-weeks-old wild-type (WT) and cytokinin double-receptor mutants (*cre1 ahk2* and *cre1 ahk3*) after infection with *B. cinerea*. Inoculated leaves were detached for photographing at 2 days post inoculation (dpi). Scale bars = 1 cm. **(B)** Disease lesion size in the indicated genotypes. The values shown are the means ± SD (*n* = 50 inoculated leaves). **(C)** The qPCR analysis of *B. cinerea* biomass in the leaves of indicated *Arabidopsis* genotypes. Data shown are the mean values ± SD (*n* = 3 biological replicates). In **(B,C)**, mean values with statistically significant differences are indicated by asterisks (two-tailed Student’s *t*-test: **P < 0.01; ***P < 0.001).

To further confirm the importance of CK in the plant–pathogen interaction between *Arabidopsis* and *B. cinerea*, CK double-receptor mutants (*cre1 ahk2* and *cre1 ahk3*) were inoculated with *B. cinerea* spores. Disease symptoms were recorded 48 h post-inoculation by measuring their lesion size (diameter) and fungal development with real-time PCR analysis (Figure 4). These results revealed larger lesions and enhanced fungal growth in *cre1 ahk2* and *cre1 ahk3* mutants than the wild-type, indicating that those plants with repressed CK signaling were more susceptible to *B. cinerea*.

**FIGURE 5:**
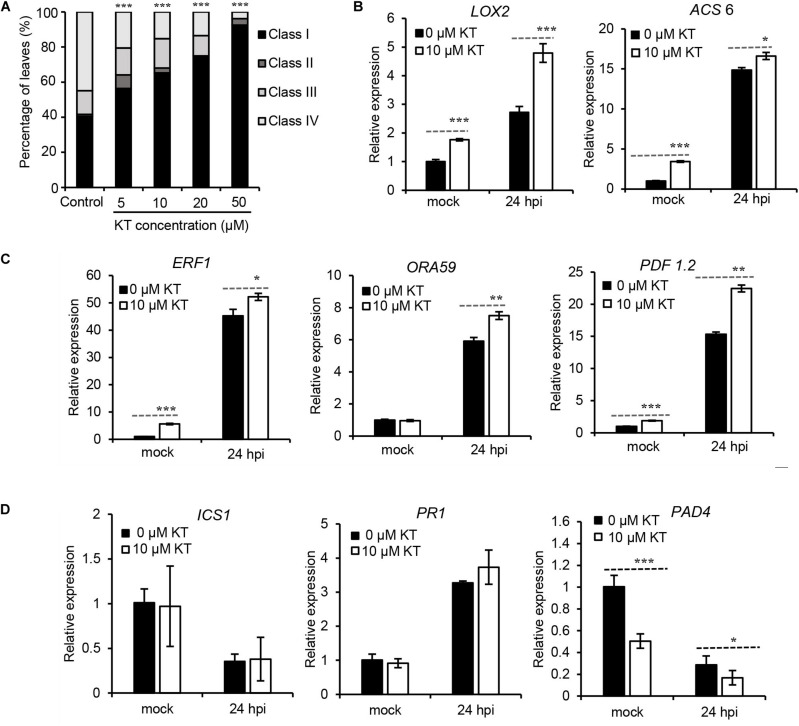
Pre-treatment of kinetin increased *Arabidopsis* resistance to *Botrytis cinerea* and transcript levels of JA/ET related genes. **(A)** Disease symptoms of leaves from 4-weeks-old *Arabidopsis* pre-treated with different concentrations of kinetin (KT) for 3 days before 2 days *B. cinerea* infection. Class I, lesion < 2 mm; Class II, 2 mm lesion plus chlorosis; Class III, lesion 2–4 mm plus chlorosis; Class IV, lesion > 4 mm plus chlorosis. The distribution at each kinetin concentration was calculated from 50 leaves. The significance of differences was analyzed by χ^2^-test. ***P < 0.001. **(B)** Transcript levels of the JA biosynthesis gene *LOX2* and ET biosynthesis gene *ACS6*, in response to *B. cinerea* infection following prior treatment with 0 or 10 μM of KT. **(C)** Differential expression of plant immunity JA/ET-responsive genes *ERF1*, *ORA59*, and *PDF1.2* in response to *B. cinerea* infection following the 0 or 10 μM KT treatment. **(D)** Transcript levels of SA biosynthesis gene *ICS1*, SA-responsive marker gene *PR1*, and SA signaling gene *PAD4*, in response to *B. cinerea* infection following prior treatment with 0 or 10 μM of KT. In **(B–D)**, expression levels in the mock leaves at 24 h post-inoculation (hpi) with 0 μM KT pre-treatment were set to a value of 1. Error bars are standard deviations (*n* = 3). Mean values with statistically significant differences are indicated by asterisks (two-tailed Student’s *t*-test: *P < 0.05; **P < 0.01; ***P < 0.001). Three independent experiments were performed with a similar outcome; results from one representative experiment are shown.

Then, a series of concentrations of exogenous kinetin (KT, a type of cytokinin) were sprayed onto 4-weeks-old *Arabidopsis* leaves, for 3 days, before their inoculation with *B. cinerea*, followed by an assessment of disease symptoms 2 days later. Results in Figure 5A clearly illustrate that plants pre-treated with concentrations from 5 to 50 μM KT developed significantly less-severe disease symptoms than did mock-treated plants, suggesting that enhanced resistance of *Arabidopsis* to *B. cinerea* could be induced by a CK pre-treatment. To investigate whether exogenous CK application could change this susceptibility by influencing the JA/ET and SA pathways, expressions of JA/ET, SA biosynthesis genes and JA/ET-, SA-responsive genes were tested via qRT-PCR in the leaves infected with *B. cinerea*, with or without a 10-μM KT pre-treatment. As Figure 5B reveals, *LOX2* (*LIPOXYGENASE 2*), encoding a key enzyme in the octadecanoid pathway leading to JA biosynthesis, and *ACS6* (*ACC Synthase 6*), the gene product of which functions in the biosynthesis of the ethylene precursor aminocyclopropane carboxylase (ACC), were both transcriptionally upregulated by the KT pre-treatment. Further, ETHYLENE RESPONSE FACTOR1 (ERF1) and OCTADECANOID-RESPONSE ARABIDOPSIS 59 (ORA59) function as integrators of JA and ET signaling in plant defense responses (Pre et al., 2008), in which PLANT DEFENSIN1.2 (PDF1.2) acts downstream of ERF1/ORA59 and operates as part of the JA-ET-responsive antifungal defense (Spoel et al., 2003). For all three genes, their expression levels were strongly increased after applying the KT pre-treatment (Figure 5C). Taken together, these results indicated that an exogenous CK pre-treatment could promote JA/ET’s biosynthesis and response, pointing to potential crosstalk between the CK pathway and JA/ET in plant immunity. However, as seen in Figure 5D, *ICS1* (*isochorismate synthase 1*), encoding a key enzyme in SA production, and *PR1* (*PATHOGENESIS RELATED PROTEIN 1*), the SA-responsive marker gene, were both not significantly regulated by the KT pre-treatment, whereas *PAD4* (*PHYTOALEXIN DEFICIENT 4*), a lipase-like gene important for SA signaling, was transcriptionally repressed. Since the role of PAD4 in resistance to *B. cinerea* might be more complicated than that depending on its role as regulator of SA signaling pathways (Nandi et al., 2005), more work will be necessary to show CK-SA crosstalk in the plant immunity to *B. cinerea*.

### JA and ET Affect CK Levels and Signaling Differentially in the Interaction Between *Arabidopsis* and *B. cinerea*

**FIGURE 6:**
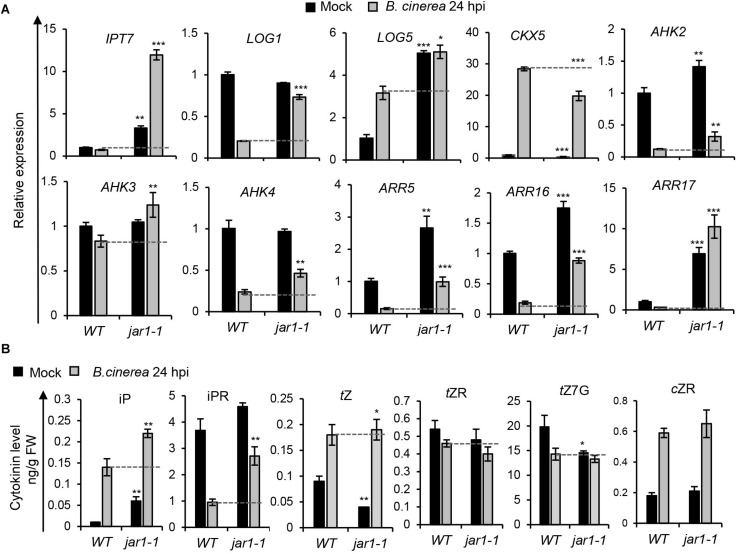
*Botrytis cinerea* infection induced cytokinin levels and signaling were regulated in the JA biosynthesis mutant. **(A)** Transcript levels of cytokinin-related genes were analyzed in leaves of the wild-type (WT) and *jar1-1* at 24 h post-inoculation (hpi) with *B. cinerea* or mock solution. The expression values in WT leaves inoculated with mock solution were set to 1. All data were normalized to the expression of *EXP* (At4g26410). Error bars represent means ± SD (*n* = 3). Three independent experiments were performed with a similar outcome; results from one representative experiment are shown. Asterisks indicate significant differences between WT and mutants with same treatments (two-tailed Student’s *t*-test, *P < 0.05; **P < 0.01; ***P < 0.001). **(B)** Cytokinin contents in leaves of WT and *jar1-1* at 24 hpi of *B. cinerea* or mock solution. Isopentenyladenine (iP), isopentenyladenosine (iPR), trans-zeatin (*t*Z), *trans*-zeatin riboside (*t*ZR), *trans*-zeatin N^7^-glucoside (*t*Z7G), and *cis*-zeatin riboside (*c*ZR) levels were measured. Bars represent means ± SD (*n* = 3). Three independent experiments were performed. Asterisks indicate significant differences between WT and mutants for the same treatment (two-tailed Student’s *t*-test, *P < 0.05; **P < 0.01). FW, fresh weight.

**FIGURE 7:**
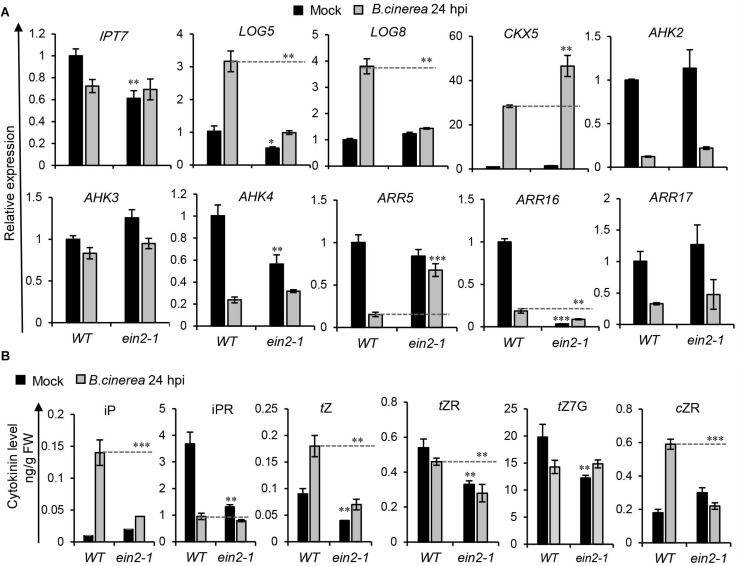
Cytokinin contents and signaling induced by *Botrytis cinerea* were changed in the ET mutant. **(A)** Transcript levels of cytokinin-related genes were measured in leaves of the wild-type (WT) and *ein2-1* at 24 h post-inoculation (hpi) of *B. cinerea* or mock solution. Expression values in WT leaves inoculated with the mock solution were set to a value of 1. All data were normalized to the expression of *EXP* (At4g26410). Bars are the means ± SD (*n* = 3). Three independent experiments were performed with a similar outcome; results from one representative experiment are shown. Asterisks indicate significant differences between WT and mutant plants with same treatments (two-tailed Student’s *t*-test, **P < 0.01; ***P < 0.001). **(B)** Cytokinin contents in leaves of WT and *ein2-1* at 24 hpi of *B. cinerea* or mock solution. Isopentenyladenine (iP), isopentenyladenosine (iPR), trans-zeatin (*t*Z), *trans*-zeatin riboside (*t*ZR), *trans*-zeatin N^7^-glucoside (*t*Z7G), and *cis*-zeatin riboside (*c*ZR) levels were measured. Error bars represent means ± SD (*n* = 3). Three independent experiments were performed. Asterisks indicate significant differences between WT and mutant plants under the same treatment (two tailed Student’s *t*-test, **P < 0.01; ***P < 0.001). FW, fresh weight.

To investigate how JA/ET may influence CK levels or signaling in the *Arabidopsis* response to *B. cinerea*, expression levels of CK-related genes and CK contents in *B. cinerea*-infected leaves were analyzed in the JA biosynthesis mutant *jar1-1* and the ET-insensitive mutant *ein2-1* (Figures 6, 7). As shown in Figure 6A, the *jar1-1* mutant’s leaves showed more pronounced transcript accumulations of *AHK2*, *AHK3*, and *CRE1/AHK4*, *ARR5*, *ARR16*, and *ARR17* at 24 hpi compared with the wild type, suggesting that impaired JA biosynthesis released CK signaling in *Arabidopsis*. Higher transcript levels of CK biosynthesis gene *IPT7*, *LOG1*, and *LOG5*, coupled to a lower transcript level of the CK oxidase gene *CKX5*, indicated that CKs’ contents might be reduced in *jar1-1*. Figure 6B and Supplementary Table S2 report the contents of iP, iPR, *t*Z, *t*ZR, *t*Z7G (*trans*-zeatin N^7^-glucoside), and *c*ZR in the wild-type and infected leaves of *jar1-1*; compared to the former plants, the *jar1-1* leaves at 24 hpi of *B. cinerea* had substantially higher levels of iP, iPR, and *t*Z, whereas no differences were observed for *t*ZR, *t*Z7G, and *c*ZR. These results suggested a possibly negative role of the JA pathway acting on CK biosynthesis and signaling during *B. cinerea* infection.

The effect of mutation in the ET pathway on CK levels and signaling are shown in Figure 7. Evidently, CK metabolism-related genes were affected by the ET pathway (Figure 7A). Specifically, at 24 hpi, the abundance of *IPT7* transcript was slightly lower in the *ein2-1* mutant than the wild type in the absence of *B. cinerea* infection; *LOG5* and *LOG8* transcript levels were strongly repressed in *ein2-1* leaves upon challenging them with *B. cinerea*; and the degradation gene *CKX5* was expressed much higher in leaves of *ein2-1* than in those of wild-type plants when inoculated with *B. cinerea*, together suggesting that CK contents might be suppressed in *ein2-1* after *B. cinerea* infection. The responses of CK-signaling genes to *B. cinerea* infection were also changed in the *ein2-1* mutant. Compared with the wild type’s infected leaves, *AHK4* and *ARR16* transcript levels were significantly repressed and *ARR5*’s expression was much higher in the infected *ein2-1* leaves (Figure 7A). These results suggested a more complex regulation mechanism underpinning the ET pathway’s influence on CK signaling during *B. cinerea* infection.

We next measured the CKs’ contents at 24 hpi in leaves of wild-type and *ein2-1* mutant inoculated with *B. cinerea* or the mock treatment. As shown in Figure 7B and Supplementary Table S2, in locally infected leaves of the *ein2-1* mutant, their iP, *t*Z, *t*ZR, and *c*ZR contents were dramatically decreased compared with the wild type. Furthermore, without *B. cinere* applied, the iPR content of the *ein2-1* mutant was lower than in the wild type, indicating that mutation in the ET pathway repressed CK biosynthesis.

**FIGURE 8:**
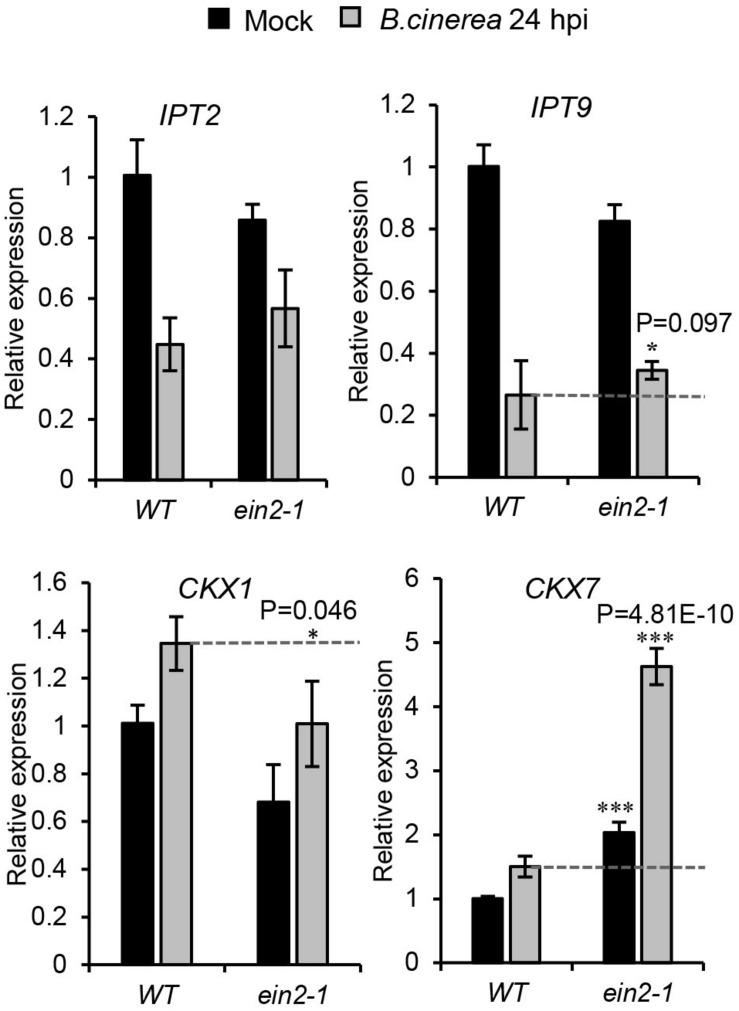
Expression profiles of genes related to *cis*-zeatin-type cytokinins’ biosynthesis and metabolism in the wild-type (*WT*) and *ein2-1* leaves drop-inoculated with or without *Botrytis cinerea* spores. Leaves were harvested at 24 h after treatments. The expression values in WT leaves with mock treatment were set to a value of 1. All data were normalized to the expression of *EXP* (At4g26410). Bars are the mean ± SD from three independent experiments. Asterisks indicate significant differences between WT and mutant plants with the same treatments (two tailed Student’s *t*-test, *0.01 < *P* < 0.05; ***P < 0.001).

Combining the Figure 3 results, which demonstrated that *c*Z-type CK contents were strongly elevated after *B. cinerea* infection, with those of Figures 6B, 7B, it appears that *c*ZR accumulation was strongly suppressed in the *ein2-1* mutant but negligibly changed in the *jar1-1* mutant. Expression levels of the genes responsible for *c*Z-type CK metabolism were further analyzed by qRT-PCR in wild-type plants and *ein2-1* mutant, with or without *B. cinerea* infection, at 24 hpi (Figure 8). This revealed that while transcript levels of *IPT9* and *CKX1* were slightly reduced after *B. cinerea* inoculation in the *ein2-1* mutant, its *CKX7*’s expression level increased remarkably. This suggested that the reduction of *c*ZR contents in *ein2-1* mutant might be driven mainly by up-regulation of *CKX7*.

## Discussion

In this paper, we addressed the responsiveness of CKs to *B. cinerea* infection in *Arabidopsis*. We showed that the CK pathway did respond to *B. cinerea* infection and preliminarily demonstrated its interactions with the JA and ET pathways. These results indicated that CKs are plant components crucially involved in their responses to the necrotrophic pathogen *B. cinerea*.

### The CK Pathway Responds to *B. cinerea* Infection

Several transcriptomics analyses of *Arabidopsis* treated with *B. cinerea* have shown that transcripts of CK-related genes are potentially regulated by *B. cinerea* infection (Argueso et al., 2012; Birkenbihl et al., 2012; Zhang et al., 2017). Here, we confirmed that *B. cinerea* infection could alter the contents of active CKs (iP, iPR, *t*Z, *t*ZR, *c*Z, and *c*ZR) in locally infected *Arabidopsis* leaves, accompanied by significant alterations of transcript levels of many genes involved in CK metabolism and signaling. We found that, from 24 hpi onward, the *B. cinerea*-infected leaves partly decayed and their contents of active *cis-*Zeatin-type CKs (*c*Z and *c*ZR) were dramatically increased, indicating a key role of *c*Z-type CKs in how *Arabidopsis* plants respond to *B. cinerea* infection (Figure 3). The *cis*Z-type CKs are thought to act as sensitive regulators of CK responses in plants under growth-limiting conditions and are able to modulate plant defense responses (Gajdošová et al., 2010; Schäfer et al., 2015a). Several pathogens have been identified which produce *c*Zs to modulate their hosts’ physiology for their own benefits (Schäfer et al., 2015a; Han and Kahmann, 2019). Besides pathogens, there is also evidence that *c*Z-type CKs are also involved in plant–herbivore interactions; for example, *Manduca sexta* and *Tupiocoris notatus* herbivory has been shown to increase the levels of *c*Z-type CKs in *N. attenuata* (Schäfer et al., 2015c; Brütting et al., 2018). Furthermore, applying *c*ZR to *N. attenuate* leaves increased MeJA-mediated induction of defense metabolites, such as those associated with the phenolamide pathway and trypsin proteinase inhibitor activity, suggesting that *c*Zs are potentially involved in defense metabolite accumulation after herbivore attack (Schäfer et al., 2015a). Since *B. cinerea* cannot produce CKs (Cooper and Ashby, 1998; Walters and McRoberts, 2006), the significant increase in levels of *c*Z-type CKs we found in the infected *Arabidopsis* leaves (Figure 3) led us to suppose that, to protect themselves, plants might manipulate the content of *c*Z-type CKs to delay the leaf senescence and defend against *B. cinerea* attacks (Swartzberg et al., 2008). Further experiments that use *Arabidopsis* plants with altered *c*Z-type CKs levels, for example, via impaired *c*Z-biosynthesis (*ipt2 9* mutants, Miyawaki et al., 2006) or increased *c*Z-degradation (*AtCKX7* overexpression, Köllmer et al., 2014) or an overproduction of *c*Z (via overexpression of *c*Z biosynthesis gene *AtIPT2*), are needed to elucidate the role of *c*Z-type CKs in *Arabidopsis* responses to *B. cinerea*.

Connecting specific alterations in transcript levels of genes involved in CK metabolism with concomitant alterations in CK contents upon the infection of *B. cinerea* is an intricate task. Since CKs have essential roles in leaf senescence, apoptosis, immunity, and complicated forms with differentiated functions (reviewed by Kieber and Schaller, 2014), during *Arabidopsis*–*B. cinerea* interactions, CK metabolism might be manipulated by the pathogen and the host in different ways for their own benefit. The transcript levels of CK-related genes could be influenced by pathogen effectors (Hann et al., 2014; Kazan and Lyons, 2014), other induced phytohormones involved in the plant defense response (Naseem et al., 2014), and tissue disruption and CK-mediated feedback regulation (Brenner et al., 2012). More genetic and biochemical experiments are needed to clearly address how CK contents and signaling are changed during the process of *B. cinerea* infection of plants.

### CK Cross Talk With JA and ET Pathways After *B. cinerea* Infection

CKs can intercommunicate with other phytohormones, including auxins, abscisic acid, and gibberellins, in the modulation of plants’ development and adaptation to stress (Seif El-Yazal et al., 2015). Defense responses to attacks by necrotrophic pathogens and herbivorous insects are considered as mainly mediated by the JA pathway acting together with ET in a coordinated manner. A few reports have investigated CK-JA interplay in plants under conditions of wounding and herbivory (Sano et al., 1996; Dervinis et al., 2010; Schäfer et al., 2015b,c), yet little is known about the possible CK-JA cross talk occurring in plant defense upon challenge with pathogens. As to the interaction between CK and the ET pathway, the correlation between CK and ET in root development or plant response to light or cold stress has been demonstrated (Shi et al., 2012; Zdarska et al., 2015; Liu J. et al., 2017), but how CK and ET interact under biotic stress conditions, including necrotroph infection, is not clear. Here we found that a pre-treatment with kinetin could strongly elevate the transcript levels of JA/ET biosynthesis genes as well as JA/ET response genes (Figures 5B,C), suggesting the positive effect of CKs on the JA/ET pathway. This could explain the *Arabidopsis* resistance to *B. cinerea* induced by the exogenous kinetin pre-treatment, which agrees with the reports that the senescence-inducible expression of a CK biosynthesis gene, *IPT*, could suppress *B. cinerea*-induced disease symptoms. The effects of kinetin upon the JA pathway in our study are also consistent with the findings of positive effects of CKs on JA levels and JA-mediated defenses to herbivores (Dervinis et al., 2010; Schäfer et al., 2015b).

Furthermore, we investigated the effects of JA and ET pathways on CK signaling and levels upon *B. cinerea* challenge at the 24-hpi time-point (Figures 6, 7). In the JA biosynthesis mutant, *jar1-1*, transcript levels of genes involved in CK signaling (including the three CK receptor genes and type-A ARR genes) were much higher in locally *B. cinerea*-infected leaves than that in mock leaves, which indicated that the repressed JA response could release the CK signaling. Accordingly, we speculated that during *B. cinerea* infection, the JA pathway may negatively affect CK signaling. The transcript levels of CK metabolism genes in the *jar1-1* mutant upon pathogen attack could be associated with changed CK levels (Figure 6). CKs contents in the *jar1-1* mutant during *B. cinerea* infection (Figure 6B) indicate a suppressive effect of JA on the iP, iPR, and *t*Z accumulation. JAs have been shown to counteract activities of CKs more generally than what our data here suggest. JAs can reduce the transcripts of CK-responsive genes (Brenner et al., 2012), and their effects are opposite those of CKs on various physiological traits, such as senescence (Richmond and Lang, 1957; He et al., 2002), leaf growth, and cell division (Noir et al., 2013; Attaran et al., 2014). In our study, the *c*ZR contents were similar between *jar1-1* and the wild type after *B. cinerea* inoculation (Figure 6B), suggesting that the JA pathway is likely not responsible for the accumulation of *cis*Z-type CKs during *B. cinerea* infection in wild-type leaves. However, this contradicts the findings in *N. attenuata*, in which JA supplementation promoted the accumulation of *c*ZR and the silencing of *COI1*, a receptor gene in JA signaling, strongly reduced *c*ZR levels, in response to *M. sexta* oral secretions (Schäfer et al., 2015c). These data indicate that JA-CK cross talk behaves differently in *Arabidopsis* and *N. attenuata*.

Different from the situations in *jar1-1*, CK contents (iP, iPR, *t*Z, *t*ZR, and *c*ZR) were all significantly reduced, biosynthesis genes (*IPT7*, *LOG5*, *LOG8*) were downregulated, and the CK degradation gene *CKX5* was upregulated in the infected leaves of *ein2-1* when compared to wild-type leaves (Figure 7). These results suggest that an induced accumulation of CKs in response to *B. cinerea* infection depended on the ET pathway. Expression of the type-A response regulator gene *ARR16* was strongly repressed, but the transcription of *ARR5* was upregulated during *B. cinerea* infection in *ein2-1*, showcasing the complexity characterizing the regulation of CK signaling by the ET pathway. In root development, the ET signaling pathway involves different receptor clusters having multiple layers of complexity in cross talk with CKs (Liu J. et al., 2017). Ethylene can both positively and negatively regulate CK signaling respectively through ARR5 and ARR2. EIN3 negatively regulates *ARR5* in *Arabidopsis* and downstream ET signaling can positively regulate the CK pathway, in turn modulating the expression of *ARR5*. In a different way, another ET receptor, ETR1, which has histidine kinase activity, can positively regulate general cytokinin signaling through ARR2, which upregulates CK oxidase. Most recently, ETR1 was shown to regulate CK signaling specifically in the root transition zone, presumably via regulation of *ARR10*, a positive regulators of the multistep phosphorelay (MSP) pathway, which differs from canonical CTR1/EIN2/EIN3 ethylene signaling and is independent of EIN2 (Zdarska et al., 2019). We find that JA and ET can regulate CK signaling and levels in a rather different manner after *B. cinerea* infection, though JA and ET pathway are reportedly able to also mediate plant defense responses in a synergistic way (AbuQamar et al., 2017).

**FIGURE 9:**
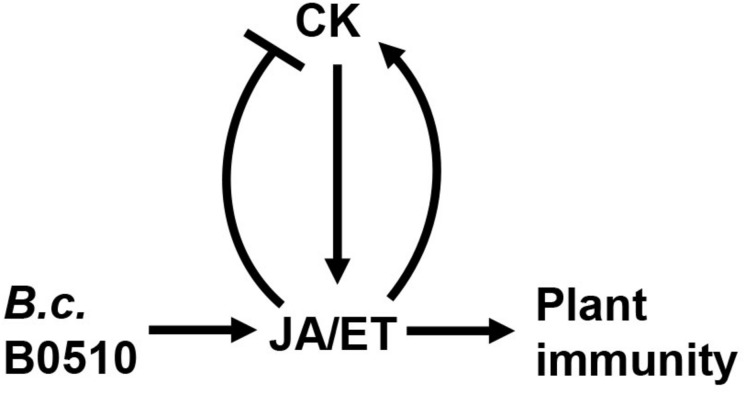
Proposed model showing how CK and JA/ET pathways interact during *B. cinerea* infection. Upon *B. cinerea* infection, JA and ET pathways are activated. CK promote resistance of *Arabidopsis* to *B. cinerea* by positively regulating JA and ET pathway. Conversely, JA negatively regulates CK signaling and content, while ET interacts positively with the CK pathway.

Taken together, CK signaling and levels were modulated after *B. cinerea* infection. CK signaling was repressed, but *cis*-Zeatin-type CKs (*c*Z and *c*ZR) contents were strongly increased in locally infected *Arabidopsis* leaves. Both the JA and ET pathways contribute to changed CK signaling and levels during *B. cinerea* infection, albeit differently. Based on our results, we propose that the JA pathway can adversely affect CK signaling and the production of iP, iPR, and *t*Z upon *B. cinerea* challenge, whereas the ET pathway affects the active CKs’ contents in a positive way following infection by this fungus (Figure 9). Clarifying how JA and ET pathways regulate the response of CKs to *B. cinerea* will provide new insights into these complex interactions occurring on the battlefield between plant and *B. cinerea*.

### CK Cross Talk With the SA Pathway After *B. cinerea* Infection

The function of SA in immune responses to *B. cinerea* may vary, depending on the plant species. In tomato, applying SA significantly increases its resistance to *B. cinerea* (Angulo et al., 2015). By contrast, in *Arabidopsis*, an increase in SA level has either no or only affects resistance at the primary infection site (Ferrari et al., 2003). Tomato *NahG* plants overexpressing an SA hydroxylase to decrease SA levels *in vivo* showed high susceptibility to *B. cinerea*, while tobacco and *Arabidopsis NahG* plants responded similarly to the wild type to the same pathogen (Ferrari et al., 2003; Asai et al., 2010; El Oirdi et al., 2011). In plants, SA can antagonize the JA signaling pathway and *vice versa*. Since *B. cinerea* can produce an exopolysaccharide as an elicitor of the SA pathway, this activated SA pathway antagonizes the JA signaling pathway through NPR1, enabling the pathogen to promote disease development in tomato (El Oirdi et al., 2011). The CK pathway has been proven to have a role in SA-JA cross talk. ARR11, the B-type response regulator of CKs, has been described as a novel negative regulator of SA-JA cross talk, promoting its resistance against *B. cinerea* (Proietti et al., 2018). Moreover, CK can strengthen the immunity of *Arabidopsis* to hemi-biotrophs via ARR2, by interacting with the SA transcription factor TGA3 (Choi et al., 2010). In our study here, the KT pre-treatment did not significantly change the transcript levels of SA biosynthesis gene *ICS1* or the SA-responsive marker gene *PR1* during *B. cinerea* infection, but it suppressed the level of another SA signaling gene, *PAD4* (Figure 5D). In tomato leaves, however, an external CK pre-treatment led to greater internal SA production and their increased resistance to *B. cinerea* due to CK pre-treatment being SA- and ET-dependent, but the changes in the JA content and pathway were not described (Gupta et al., 2020). Lower SA levels may be correlated with increased *B. cinerea* resistance in some cases in tomato (Angulo et al., 2015; Mehari et al., 2015), and Gupta et al. (2020) proposed that the severity of *B. cinerea* disease and the resultant increase in SA levels depend on both host and the pathogen isolate. In plant–*B. cinerea* interactions, the cross talk that occurs among the phytohormones CK, SA, and JA/ET seems highly complex and a puzzle that can only be solved with more concerted research efforts.

## Data Availability Statement

The original contributions presented in the study are included in the article/Supplementary Material, further inquiries can be directed to the corresponding author/s.

## Author Contributions

LC designed the study. LC, BL, RW, and SW performed the experiments and analyzed the data. LC and JZ discussed the results and wrote the manuscript. All authors contributed to the article and approved the submitted version.

## Conflict of Interest

The authors declare that the research was conducted in the absence of any commercial or financial relationships that could be construed as a potential conflict of interest.

## Footnotes

1 http://rsb.info.nih.gov/ij/

2 https://genevestigator.com/gv/
