# Supplementary material for: Diversified Regulation of Cytokinin Levels and Signaling During Botrytis cinerea Infection in Arabidopsis
Source: Front Plant Sci. 2021 Feb 10;12:584042. doi: 10.3389/fpls.2021.584042 (PMC7902887; doi:10.3389/fpls.2021.584042)
Supplement: Supplementary file 1 [file Table_1.docx]

**Supplementary information**

**
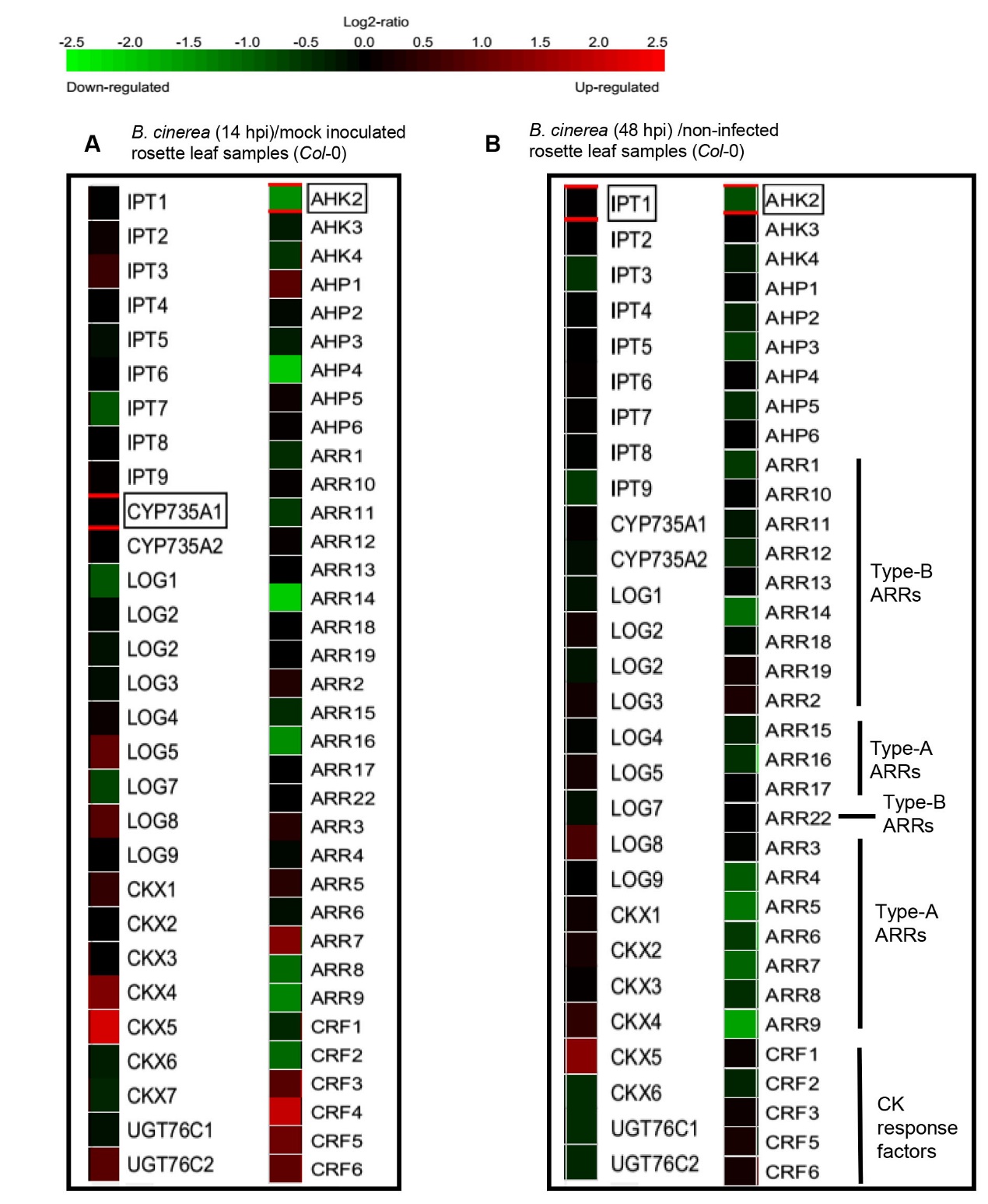
**

**Figure S1. Heat map of expression of genes involved in Cytokinin metabolism and signaling in *Arabidopsis* leaves following *Botrytis cinerea* infection.** Microarray data was obtained from GENEVESTIGATOR (<https://genevestigator.com/gv/>). *IPT* genes, *CYP735A* genes and *LOG*s are involved in CK biosynthesis. *CKX* genes encode CK degradation enzymes. *UGT76C* genes encode CK glycosyltransferases. *AHK2*, *AHK3* and *AHK4*/*CRE1* are CK receptors. AHPs are histidine phosphotransferase proteins. (A) Heat map of gene expression at 14 hours post inoculation (hpi) of *B. cinerea*. (B) Heat map of gene expression at 48 hpi.


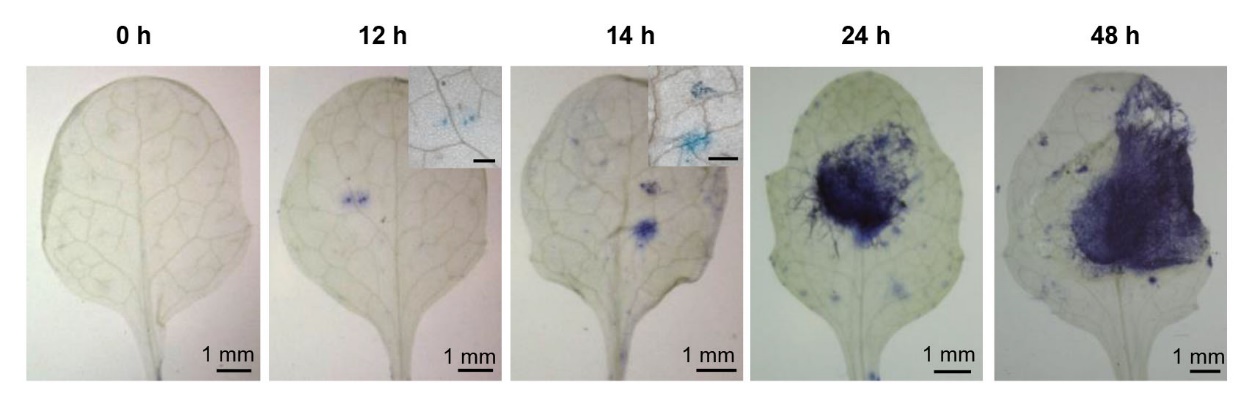


**Figure S2.** **Fungal growth on wild-type (WT) leaves at different time points after inoculation.** Leaves of 4-week-old WT plants were dropped with 4 µL 2.5*10^5^ spores mL^-1^ of *B. cinerea*. Fungal growth and cell death development was analyzed by staining with trypan blue at 0 h, 12 h, 14 h, 24 h and 48 h post inoculation. The pictures at the upper right corners of the photos at 12 h and 14 h were localized zoom in of the blue staining. The error bars for the zoom in pictures are 400 µm.

**Table S1** Concentration of endogenous cytokinins (ng/g fresh weight) in response to *B. cinerea* infection

Cytokinins

Mock

14 h

*B. Cinerea*

14 h

Mock

24 h

*B. Cinerea*

24 h

Mock

48 h

*B. Cinerea*

48 h

iP

iPR

0.09±0.01

0.11±0.01

0.05±0.01

0.14±0.02

0.06±0.01

0.66±0.02

1.61±0.09

1.55±0.08

1.46±0.15

0.75±0.14

1.87±0.12

0.95±0.07

*t*Z

0.14±0.01

0.12±0.01

0.07±0.01

0.15±0.01

0.03±0.01

0.58±0.03

*t*ZR

1.49±0.04

1.30±0.04

0.81±0.06

0.46±0.02

0.52±0.02

0.16±0.02

cZ

n.d.

0.01±0.00

n.d.

0.05±0.00

n.d.

0.72±0.02

*c*ZR

0.30±0.01

0.28±0.04

0.25±0.02

0.46±0.02

0.19±0.02

1.14±0.06

Analyzed tissue: leaves of 4-week-old Arabidopsis plants 14 h, 24 h and 48 h post inoculation of *B. cinerea* and mock solution. Shown are mean values ± SD (n=3). n.d., not detected. iP, isopentenyladenine; iPR, isopentenyladenosine; *t*Z, *trans*-zeatin; *t*ZR, *trans*-zeatin riboside; *c*Z, *cis*-zeatin; *c*ZR, *cis*-zeatin riboside.

**Table S2** Levels of cytokinins in wild type (WT), *jar1-1* and *ein2-1* 24 h after *B. cinerea* inoculation

Cytokinins

iP

iPR

0.01±0.00

0.14±0.02

0.06±0.01

0.22±0.01

0.02±0.00

0.04±0.00

3.68±0.44

0.95±0.12

4.59±0.14

2.71±0.35

1.31±0.08

0.79±0.04

*t*Z

0.09±0.01

0.18±0.02

0.04±0.00

0.19±0.02

0.04±0.00

0.07±0.01

*t*ZR

0.54±0.05

0.46±0.02

0.48±0.06

0.36±0.04

0.33±0.02

0.28±0.05

*t*Z7G

14.28±1.22

13.29±0.70

14.84±0.74

*c*ZR

0.18±0.02

0.59±0.03

0.21±0.03

0.65±0.09

0.30±0.03

0.22±0.02

WT mock

WT *B.c* treatment

*jar1-1* mock

*jar1-1* *B.c* treatment

*ein2-1* mock

*ein2-1* *B.c* treatment

19.81±2.34

14.53±0.32

12.25±0.49

Analyzed tissue: the leaves of 4-week-old WT, *jar1-1* and *ein2-1* plants 24 h after inoculation with *B. cinerea* spores suspension or mock half PDB solution. Shown are mean values ± SD (n=3). iP, isopentenyladenine; iPR, isopentenyladenosine; *t*Z, *trans*-zeatin; *t*ZR, *trans*-zeatin riboside; *t*Z7G, *trans*-zeatin N^7^-glucoside; *c*ZR, *cis*-zeatin riboside.

**Table S3.** Primers used for quantitative real-time PCR

| Gene | Forward primer (5’-3’) | Reverse primer (5’-3’) |
| --- | --- | --- |
| *EXP* | GAGCTGAAGTGGCTTCCATGAC | GGTCCGACATACCCATGATCC |
| *PDF1.2* | TGCTTCCATCATCACCCTTA | CACTTGTGTGCTGGGAAGAC |
| *ACS6* | AAACCGAACTATGGCGTGTG | TCATGGCAATGGAACGAAC |
| *IPT2* | TGGGACGGTGAGCTCAGATA | ATCTGCACAACACTCCTCGG |
| *IPT3* | TCCGTCCTAAACCGTGGAA | CCATTCCACTCTCCACCATC |
| *IPT7* | TGTAGCGAGACCAAGCGAGAGA | GTTTCATGGATTCCAGGAGCTTC |
| *IPT9* | GCTGTGGAGTTGGTGGTCAATGCT | TGCGAAAGGAAGACGGAGGCGA |
| *LOG1* | GACAGTGGGAGAAGTGAAGG | TTCAAGTGTCCCATACCCAC |
| *LOG5* | CCATCTCAGCGTCACATCTT | TTTCTTCTCAACCTCCCACC |
| *LOG7* | TAAGGCTGTGGACGAAGGA | CGGTCCACTTCATCCCAAA |
| *LOG8* | TGCTCCAACAGCCAAAGAG | CGGCTTGTTTTCTTGTCCC |
| *CKX1* | CTGAGAAGCGGAATTCTGAAC | GAGTACCCTGATCCATTTAACCA |
| *CKX4* | ATAAAGGCTCAACCAGCCCC | ACGTCATGTTCACGACGACA 434 |
| *CKX5* | ACGGACGGTGAAGAGACACAGA | CCTGTGTTGCGTGGTGAGGA |
| *CKX7* | CACCAGAGCTAGGGTTTTGC | CATCGAACTCGGTGTATACTACTCTT |
| *AHK2* | GAGCTTTTTGACATCGGG | TTCTCACTCAACCAGACGAG |
| *AHK3* | GTGACCAGGCCAAGAACTTA | CTTCCCTGTCCAAAGCAA |
| *AHK4* | AATCGCAGTTTCTTGCTACCGTGTCT | CCACATACTTGAGCGGTTTGAGCGT |
| *AHP4* | ACGGTCCAAATGTCTCTATG | TCCACTCTTTTCTGTATTCCGT |
| *ARR4* | CCGTTGACTATCTCGCCT | CGACGTCAACACGTCATC |
| *ARR5* | CTACTCGCAGCTAAAACGC | GCCGAAAGAATCAGGACA |
| *ARR6* | GAGCTCTCCGATGCAAAT | GAAAAAGGCCATAGGGGT |
| *ARR7* | CTTGGAACCAATCTGCTCTC | ATCATCGACGGCAAGAAC |
| *ARR9* | GATAGAGCACGTCCTAGATTCG | CTGCATTCCCTACTGAAACC |
| *ARR14* | TCTTCTTTCTTCTCCAACCA | TGAAGTATCATCGTCTACAAC |
| *ARR16* | GCATTGGAGTATTTGGGTTTGGGAGA | AGATTTGATGACTCCTGCTTCACTTTCTTG |

**Table S4.** Oligonucleotides used for *Botrytis cinerea* growth biomass

| Gene | Forward primer (5’-3’) | Reverse primer (5’-3’) |
| --- | --- | --- |
| *BcCutA* | GATGTGACGGTCATCTTTGCCC | CCGATCCAGACACTGTACTTCCTT |
| *Atactin* | GATGTGACGGTCATCTTTGCCC | CCGATCCAGACACTGTACTTCCTT |
